# Hypertension, preeclampsia, and HELLP syndrome in pregnancy

**DOI:** 10.2478/abm-2023-0061

**Published:** 2023-10-26

**Authors:** Editorial Office of Asian Biomedicine

**Affiliations:** 1 Faculty of Medicine, Chulalongkorn University, Bangkok 10330, Thailand

Hypertension is common during pregnancy [1]. In many patients, there is no history of previous hypertension. All pregnant women should be periodically monitored to detect the presence of hypertension, because this condition can be associated with major problems that require timely detection and management. A previously normotensive pregnant woman who develops systolic blood pressure ≥140 mmHg and/or diastolic blood pressure ≥90 mmHg on at least two occasions 4 h apart after 20 weeks of gestation should be monitored for possible eventual unwanted pregnancy outcomes.

All pregnant women with hypertension need to be tested for proteinuria and related parameters for preeclampsia-related end-organ dysfunction. These parameters include the presence of thrombocytopenia, renal insufficiency, elevated liver transaminases, pulmonary edema, and recent cerebral or visual symptoms. Those who have negative proteinuria and do not have significant evidence of end-organ dysfunction upon follow-up can be classified as having gestational hypertension. For these patients, only sufficient control of blood pressure is adequate [2].

Pregnant women with new hypertension who are tested positive for significant proteinuria or end-organ dysfunction upon follow-up are considered to have preeclampsia [2]. For these patients, an evaluation should be carried to document the presence of chronic hypertension, chronic kidney disease, pheochromocytoma, and their recent medication profiles [3]. Hypertension and proteinuria are cornerstones for the diagnosis of preeclampsia [2,3,4]. Other systemic organ dysfunctions can ensue due to autoimmune and angiogenic mechanisms. Therefore, the presence of thrombocytopenia and/or elevated transaminases may prompt further investigation for the presence of acute fatty liver in pregnancy, thrombotic microangiopathy, or autoimmune disease with potential vascular complications such as systemic lupus erythematosus (SLE), or antiphospholipid syndrome (APS), among others [3].

At the initial prenatal visits, patients should be evaluated for relevant risk factors such as past history of preeclampsia, proteinuria, chronic hypertension, chronic kidney disease, or autoimmune conditions such as APS and SLE [2]. Preeclampsia typically becomes apparent after 34 weeks of gestation and progresses until birth. Some patients may develop preeclampsia earlier in gestation, intrapartum, or postpartum [3]. Patients with preeclampsia are at increased risk for life-threatening events, including placental abruption, acute kidney injury, cerebral hemorrhage, hepatic failure or rupture, pulmonary edema, stroke, cardiac failure, and progression to eclampsia [4]. The fetus of mothers with preeclampsia is at increased risk for growth restriction and medically or obstetrically indicated preterm birth. Placental delivery always results in complete resolution of the maternal signs and symptoms of the disease over a variable period [2,3,4].

Some pregnant patients with preeclampsia present with hemolysis, elevated liver enzymes, and low platelets, or the HELLP syndrome [2]. HELLP syndrome and preeclampsia can have a familial tendency. In this regard, Nergiz et al. [5] reported a significant increase in placental Hoffbauer cells, syncytiotrophoblast cell counts as well as several ultrastructural changes that were observed in the HELLP group compared with the control group. HELLP occurs in less than 1% of pregnancies and, in most cases, coexists with preeclampsia. Both conditions show a familial tendency [6]. Both conditions are severe pregnancy-specific disorders typically, but not necessarily associated with hypertension [7]. Both are believed to be caused by the placenta and cured only by delivery [7]. Combinations of multiple gene variants, each with a moderate risk, with concurrent maternal and environmental factors are thought to be the etiological mechanisms [8]. Certain molecular advances in preeclampsia and HELLP syndrome as well as their implications on management have been discussed [6].

A better understanding of preeclampsia and HELLP syndrome is needed for timely detection, accurate diagnosis, and proper management of this life-threatening condition [2,3,4]. At the moment, women with a high risk of developing this entity are those with a history of HELLP or preeclampsia in previous pregnancies. While a single genetic cause for the increased risk of HELLP or preeclampsia cannot be identified, multiple gene variants plus concurrent maternal and environmental factors may help early identification of the conditions to avoid unwanted complications. Meanwhile, pregnant women should be frequently monitored for abnormal blood pressure, proteinuria, and related parameters for preeclampsia-related end-organ dysfunction such as thrombocytopenia, renal insufficiency, elevated liver transaminases, pulmonary edema, and cerebral or visual symptoms. If complications occur, an induction of labor or caesarean section for early delivery can reverse the condition [8].
